# Strong associations of serum selenoprotein P with all-cause mortality and mortality due to cancer, cardiovascular, respiratory and gastrointestinal diseases in older German adults

**DOI:** 10.1007/s10654-023-01091-4

**Published:** 2024-01-10

**Authors:** Ben Schöttker, Bernd Holleczek, Sandra Hybsier, Josef Köhrle, Lutz Schomburg, Hermann Brenner

**Affiliations:** 1 Division of Clinical Epidemiology and Aging Research, German Cancer Research Center, Im Neuenheimer Feld 581, 69120 Heidelberg, Germany; 2 Saarland Cancer Registry, Neugeländstraße 9, 66117 Saarbrücken, Germany; 3 Institut für Experimentelle Endokrinologie, Max Rubner Center (MRC) for Cardiovascular Metabolic Renal Research, Charité University Medicine Berlin, CCM, Hessische Straße 4A, 10115 Berlin, Germany; 4 Division of Preventive Oncology, German Cancer Research Center, Im Neuenheimer Feld 460, 69120 Heidelberg, Germany

**Keywords:** Selenium, Selenoprotein P, Biomarker, Ageing, Mortality, Cohort study

## Abstract

**Background:**

Selenium is an essential trace mineral. The main function of selenoprotein P (SELENOP) is to transport selenium but it has also been ascribed anti-oxidative effects.

**Methods:**

To assess the association of repeated measurements of serum SELENOP concentration with all-cause and cause-specific mortality serum SELENOP was measured at baseline and 5-year follow-up in 7,186 and 4,164 participants of the ESTHER study, a German population-based cohort aged 50–74 years at baseline.

**Results:**

During 17.3 years of follow-up, 2,126 study participants (30%) died. The relationship of serum SELENOP concentration with all-cause mortality was L-shaped, with mortality being significantly higher at SELENOP concentrations < 4.1 mg/L, which is near the bottom tertile’s cut-off (4.2 mg/L). All-cause mortality of participants in the bottom SELENOP tertile was significantly increased compared to subjects in the top tertile (hazard ratio [95% confidence interval]: 1.35 [1.21–1.50]). SELENOP in the bottom tertile was further associated with increased cardiovascular mortality (1.24 [1.04–1.49]), cancer mortality (1.31 [1.09–1.58]), respiratory disease mortality (2.06 [1.28–3.32]) and gastrointestinal disease mortality (2.04 [1.25–3.32]). The excess risk of all-cause mortality for those in the bottom SELENOP tertile was more than twice as strong in men as in women (interaction of SELENOP and sex; *p* = 0.008).

**Conclusions:**

In this large cohort study, serum SELENOP concentration was inversely associated with all-cause and cause-specific mortality. Consistent inverse associations with multiple mortality outcomes might be explained by an impaired selenium transport and selenium deficiency in multiple organs. Trials testing the efficacy of selenium supplements in subjects with low baseline SELENOP concentration are needed.

**Trial registration:**

Retrospectively registered in the German Clinical Trials Register on Feb 14, 2018 (ID: DRKS00014028).

**Supplementary Information:**

The online version contains supplementary material available at 10.1007/s10654-023-01091-4.

## Background

Selenium is an essential trace mineral and has physiological functions mainly through its incorporation in selenoproteins [1]. Selenoprotein P (SELENOP) is the most abundant circulating selenoprotein and accounts for more than 50% of selenium in blood [2, 3]. The other major selenoprotein in blood (20–30%) is extracellular glutathione peroxidase (GPX) 3 [2, 3]. Furthermore, plasma proteins can contain selenium in the form of unspecifically-inserted selenomethionine, in particular albumin, which accounts for 10–20% of selenium in blood [2, 3]. The main function of SELENOP is to transport selenium throughout the body, and to supply the essential trace element to the different tissues in a hierarchical manner, thereby contributing to the biosynthesis of selenoproteins implicated in the antioxidative defence [4]. SELENOP is not only a sensitive biomarker for selenium status at low-to-moderate selenium intakes [5], but constitutes also a functional biomarker of selenium supply due to its receptor-specific uptake at target sites [6].

Low intake of selenium leads to poor expression of enzymes that are low in the hierarchical order of selenoproteins, among which intracellular GPX1 and extracellular GPX3 are most sensitive to selenium deficiency [7–9]. Mice with impaired GPX1 gene expression have high amounts of peroxidized lipids in the liver and an increased release of hydrogen peroxide into the circulatory system [10]. There is emerging evidence that GPX1 is an indispensable cornerstone of the human antioxidant defense system [11]. Impaired expression of kidney-derived GPX3 has been associated with e.g. promoting platelet-dependent arterial thrombosis [12], amyotrophic lateral sclerosis [13] and cancer [14]. The anti-oxidative functions of the GPX family members and other selenoproteins may have an impact on the lifespan [15, 16]. According to the free radical theory of ageing, an imbalance of oxidative and anti-oxidative capacities in cells can lead to damage to DNA, proteins and membrane lipids, which can result in cell senescence, the manifestation of age-related diseases and ultimately a premature death [17, 18].

In contrast to the USA, European countries have low-to-moderate dietary selenium supply due to lower selenium intake, which is caused by both a lower selenium concentration in soil (and subsequently in plants and meat as the main dietary selenium sources) and a lower use of dietary supplements [19–21]. Whereas with the National Health and Nutrition Examination Survey (NHANES) a large study about selenium status and mortality in the general adult population is available from the USA [22, 23], we conducted the largest study on this topic from Europe so far. The primary aim of this study was to assess whether the serum SELENOP concentration is inversely associated with all-cause and cause-specific mortality in a large cohort of older adults from Germany. The secondary aim of this study was to identify the determinants of SELENOP concentrations both in a cross-sectional and longitudinal study design, which was never done before. These additional analyses also have the purpose of finding the optimal set of covariates in the survival models.

## Methods

### Study design

This investigation is based on the ESTHER study (Full German name: “Epidemiologische Studie zu Chancen der Verhütung, Früherkennung und optimierten Therapie chronischer Erkrankungen in der älteren Bevölkerung”), an ongoing cohort study, details of which have been reported elsewhere [24, 25]. Briefly, 9,940 men and women, aged 50–75 years at baseline, were recruited by their general practitioners (GPs) during a routine health check-up between 07/2000 and 07/2002 in the German federal state Saarland. This health check-up is offered every two years to all Germans aged 35 years and older free of charge for all with statutory health insurance and people with private health insurance usually get the costs reimbursed. In the age range of the ESTHER study (50–75 years), differences in the utilization of the health check-up according to sex and socioeconomic status are very small [26], speaking against a selection bias by health-conscious behavior. Furthermore, the prevalences of common chronic diseases in the ESTHER study, like hypertension and diabetes mellitus, were similar to those reported for comparable age groups by the population-based German National Health Interview and Examination Survey 1998 (BGS98) [27, 28], which is a representative survey for Germany. The actual participation rate of the ESTHER study at baseline is unknown because the GPs did not document how many potential study participants they approached. We assume that it is much higher than the participation rate of the BGS98, which was 61.4% [28], as the ESTHER participants were not sent impersonal letters but were approached about the study by their familiar GP while they were in the GP’s practice for the health check-up anyway.

The ESTHER study participants have been re-contacted by mail every 2–3 years for 20 years so far. They provided detailed information about their health status in questionnaires and blood samples and since the 5-year follow-up, they donated further blood samples at their GP’s practices. Provided blood samples were centrifuged, shipped to the study center, and stored at -80 °C. Since the 8-year follow-up, the GPs also fill questionnaires about the health status of their patients because with ongoing follow-up more and more study participants reach an age-appropriate state of health in which they cannot complete questionnaires. The response rates in the follow-ups among survivors with health-related information about study participants by either the participant’s or GP’s questionnaire were 96%, 88%, 78%, 66%, 59%, and 58% for the 2-, 5-, 8-, 11-, 14-, and 17-year follow-up (data cleaning of the 20-year follow-up is pending).

### Assessment of covariates

This analysis uses data from the baseline assessment and the 5-year follow-up, in which 88% participated again. Information on socio-demographic characteristics, school education, lifestyle and diet were obtained by a comprehensive questionnaire from the study participants at baseline and at 5-year follow-up. At baseline, height, weight, systolic blood pressure, hypertension, coronary heart disease, dyslipidaemia and diabetes mellitus were assessed and documented on a standardized form by the GPs during the health check-up. At 5-year follow-up, weight and diseases were self-reported and the latter were validated by inquiry at the study participant’s GPs. Drugs against dyslipidaemia and diabetes mellitus were recorded to complement diagnosis information. Prevalent cardiovascular disease (CVD) was defined by physician-reported coronary heart disease, a self-reported history of myocardial infarction, stroke, or revascularisation of the coronary arteries (bypass or stent). Information on a life-time history of cancer (ICD-10-codes C00-C97, except C44) was provided by the Saarland Cancer Registry. C-reactive protein was measured by turbidimetry at both baseline and 5-year follow-up and inflammation was defined as C-reactive protein levels ≥ 3 mg/L [29]. The total protein concentration in the serum samples was measured with the kinetic biuret method.

The automated Diasorin-Liaison analyzer (Diasorin Inc., Stillwater, USA) was employed to measure baseline 25(OH)D levels in women. The automated IDS-iSYS (Immunodiagnostic Systems GmbH, Frankfurt Main, Germany) was used to conduct the baseline measurements for the men and for both sexes at the 5-year follow-up. Both immunoassays were standardized retrospectively with the gold standard method LC-MS/MS and the details have been described elsewhere [30]. The cut-offs for vitamin D deficiency (< 30 nmol/L 25(OH)D) and vitamin D insufficiency (30–50 nmol/L 25(OH)D), proposed by the US-American Institute of Medicine (IOM), were used to define vitamin D status [31].

Creatinine levels were measured from serum samples with a kinetic Jaffé method. The estimated glomerular filtration rate (eGFR) was determined with the 2021 CKD-EPI creatinine equation [32]. Renal impairment was defined by an eGFR < 60 mL/min/1.73 m^2^.

### SELENOP measurements

All serum samples collected at baseline and 5-year follow-up were shipped to the laboratory of the Institute for Experimental Endocrinology, Charité University Medicine, Berlin. An immunometric ELISA for SELENOP quantification using monoclonal antibodies was developed [33], and used to measure the serum SELENOP concentration between 2010 and 2012. The ELISA was calibrated against the standard reference material (SRM) 1950, which was developed in an US-American sample [34] and is still the only SRM available for SELENOP measurements. A future development of a SRM with a European sample would be desirable because the European population is less heterogenous than the US-American with respect to ethnicities. In our study, the inter-assay coefficient of variation (COV) of the SELENOP assay was 4.8% for a low concentration control and 9.3% for a high concentration control. The intra-assay COV was tested against diluted human serum and was 8.0%.

The SELENOP measurements from the study participants recruited before 02/2001 had to be discarded (n = 2,587) because they correlated with the total protein concentrations of the samples (r = − 0.38, *p* < 0.001); possibly a result of different storage conditions of these samples compared to later collected ones. The SELENOP concentration of the samples collected since 02/2001 (n = 7,186) showed no correlation with the total protein concentration (r = 0.01, *p* = 0.555). From these 7,186 participants, n = 4,164 donated another blood sample at the 5-year follow-up and a repeated SELENOP measurement was conducted. In a randomly picked sample of 59 study participants, the serum selenium concentration was additionally measured and the correlation with the SELENOP concentration was high: r = 0.688 (95% confidence interval (95%CI): 0.518; 0.805).

### Mortality ascertainment

Information about the vital status between 2000 and 2018 could be obtained for 99.7% of the cohort’s participants by means of inquiry at the residents’ registration offices. From local health authorities, the underlying cause of death in terms of ICD-10 codes could be obtained for 99.6% of the deceased participants.

### Statistical analyses

**Fig. 1:**
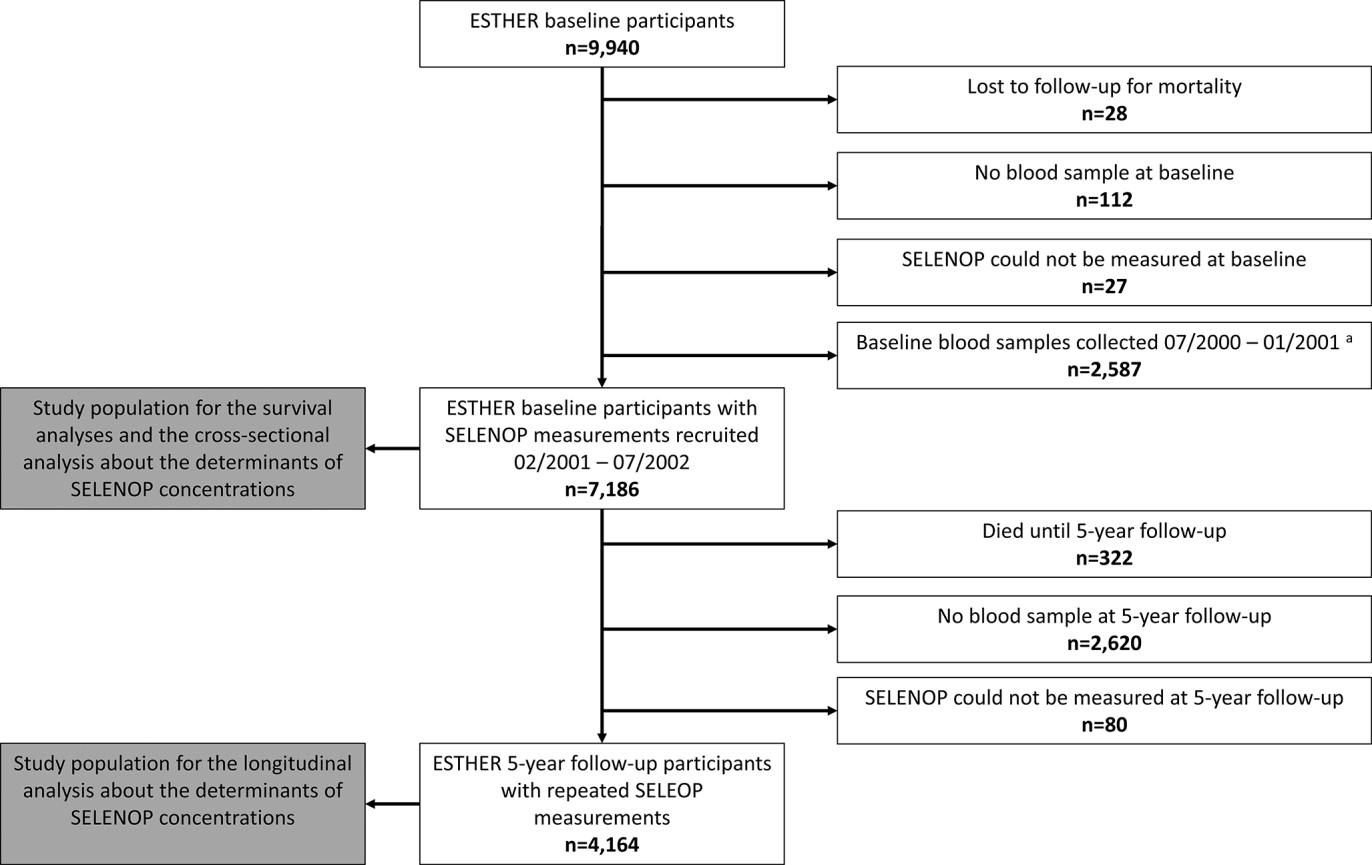
Flow-chart of the study populations utilized for the different analyses ^a^ The SELENOP measurements from the blood samples collected before 02/2001 had to be discarded because they correlated with the total protein concentrations of the samples; possibly a result of different storage conditions of these samples compared to later collected ones

Participants of the ESTHER baseline examination (n = 9,940) were excluded from this investigation if they were lost to follow-up for mortality (n = 28), provided no blood samples (n = 112) or SELENOP could not be measured (n = 27), or if they were recruited between 07/2000 and 01/2001 (n = 2,587), which resulted in a total sample size of n = 7,186 subjects for the survival analyses (see Fig. 1 for flow-chart).

To assess the determinants of a low SELENOP concentration in cross-sectional analysis, a logistic regression model with forward selection (entry criterion: *p* < 0.05) was employed with all baseline characteristics shown in Table 1 as independent variables and the baseline bottom SELENOP tertile versus the middle and top SELENOP tertiles as the dependent variable. To assess the determinants of a low SELENOP concentration in longitudinal analysis, the same model was applied but the SELENOP measurements from the 5-year follow-up were used as the dependent variable and the baseline SELENOP concentration was added as an additional independent variable. Whereas the cross-sectional analysis was conducted with all included 7,186 subjects, the longitudinal analysis was conducted with those n = 4,164 participants with repeated SELENOP measurements at the 5-year follow-up (see Fig. 1 for flow-chart).

**Table 1:** Description of the baseline characteristics of the study population

Baseline characteristics	n_total_	n (%)	Mean ± SD
Age (years)	7186		62.3 ± 6.6
Sex (Male)	7186	3219 (44.8)	
School education (years)	7000		
≤9		5264 (75.2)	
10–11		952 (13.6)	
≥12		784 (11.2)	
BMI (kg/m²)	7178		27.7 ± 4.4
< 25		1955 (27.2)	
25 - <30		3375 (47.0)	
≥ 30		1848 (25.8)	
Smoking behaviour	6989		
Never smoker		3548 (50.8)	
Former smoker		2299 (32.9)	
Current smoker		1142 (16.3)	
Physical activity ^a^	7163		
Inactive		1571 (21.9)	
Low		3281 (45.8)	
Medium or high		2311 (32.3)	
Alcohol consumption ^b^	6478		
Abstainer		2135 (33.0)	
Moderate		3909 (60.3)	
High		334 (5.2)	
Very high		100 (1.5)	
Nutrition (portions per day)			
Meat	6456		1.3 ± 0.9
Bread	5980		1.6 ± 1.2
Milk/cheese/eggs	6539		1.6 ± 1.2
Fruits/vegetables	6813		2.0 ± 1.4
Fish	6754		0.2 ± 0.2
Multivitamin/mineral supplements (daily)	7017	991 (14.1)	
Diseases/conditions			
Cancer ^c^	7186	458 (6.4)	
CVD ^d^	7185	1200 (16.7)	
Diabetes mellitus	7073	1062 (15.0)	
Dyslipidemia	7075	3100 (43.8)	
Hypertension	7071	3132 (44.3)	
Heart failure	7136	755 (10.6)	
Renal impairment ^e^	7165	1194 (16.7)	
Inflammation ^f^	7097	2768 (39.0)	
Vitamin D status ^g^	7020		50.3 ± 22.6
Sufficient		2795 (39.8)	
Insufficient		3146 (44.8)	
Deficient		1079 (15.4)	
Total protein (g/L)	7186		72.5 ± 4.9
Baseline SELENOP (mg/L)	7186		4.8 ± 1.4
5-year follow-up SELENOP (mg/L)	4164		3.9 ± 1.0

Abbreviations: BMI, body mass index; CVD, cardiovascular disease; n_**total**_, number of participants with data; SELENOP, selenoprotein P; SD, standard deviation

^a^ “Inactive”: <1 h of vigorous or light physical activity/week. “Medium or high”: ≥2 h of vigorous and ≥ 2 h of light physical activity/week. All other amounts of physical activity were grouped into the category “Low”

^b^ “Moderate”: women > 0–<20 and men > 0–<40 g ethanol per day. “High”: women ≥ 20-<40 and men ≥ 40-<60 g ethanol per day. “Very high”: women ≥ 40 and men ≥ 60 g ethanol per day

^c^ History of any cancer except non-melanoma skin cancer

^d^ CVD was defined as coronary heart disease or history of myocardial infarction or stroke

^e^ Estimated glomerular filtration rate (eGFR) < 60 ml/min/1.73 m²

^f^ C-reactive protein ≥ 3 mg/L

^g^ Vitamin D deficient, 25-hydroxyvitamin D (25(OH)D) < 30 nmol/L; insufficient, 25(OH)D 30 - < 50 nmol/L; sufficient, 25(OH)D ≥ 50 nmol/L

Cox proportional hazards models were used to estimate HRs for SELENOP tertiles (top tertile as the reference category) with respect to all-cause mortality and mortality due to CVD, cancer, respiratory diseases, gastrointestinal diseases, and psychiatric/neurological diseases, respectively. Two models were used: an age- and sex-adjusted and a comprehensively adjusted main model. The set of covariates for the main model consisted of all factors, which were both associated with the SELENOP concentration (in either cross-sectional or longitudinal analysis) as well as with all-cause mortality. If not stated otherwise, all Cox proportional hazards models used repeated SELENOP measurements and covariate assessments from the 5-year follow-up by fitting all variables of the model except sex as time-dependent variables using an addition to the SAS PHREG procedure described elsewhere [35]. For study participants who did not participate in the 5-year follow-up or who did not donate a blood sample at that follow-up, it was assumed that their baseline characteristics and their allocation to the SELENOP tertile did not change between baseline and 5-year follow-up.

The concentration-response relationship between the baseline SELENOP concentration and all-cause mortality was plotted with restricted cubic splines using Cox proportional hazards regression and the covariate set of the main model [36]. Five knots at the 10th, 25th, 50th, 75th and 90th SELENOP concentration percentile were used and the median was used as the reference.

In sensitivity analysis, only the baseline SELENOP measurements were used for all Cox proportional hazards regression models that used time-dependent modelling of the SELENOP concentration and all covariates. In a further sensitivity analysis, analyses were repeated without time-dependent modelling using either only deaths that occurred earlier (year 1–9) or later (year 10–18) during follow-up.

Subgroup analyses were carried out by sex, age groups (< / ≥ 65 years), CVD and history of cancer. Potential interactions of the SELENOP tertiles with the covariates of the main model were tested for statistical significance by adding product terms to the model.

Multiple imputation was employed to adequately deal with missing baseline and 5-year follow-up covariate values. The Markov Chain Monte Carlo (MCMC) method of the SAS procedure PROC MI was employed to impute 5 data sets. All variables shown in Table 1 were used for the imputation model. The proportion of imputed missing values varied from 0 to 16.8% (Table 1). No reasons to discard the missing at random assumption were known. All analyses were performed in the 5 imputed data sets and results of the individual data sets were combined by the SAS procedure PROC MIANALYZE.

All statistical tests were two-sided using an alpha level of 0.05 and all analyses were conducted with the software package SAS, version 9.4 (Cary, North Carolina, USA).

## Results

### Characteristics of the study population

The mean age of the 7,186 included study participants was 62.3 years and 3,219 (44.8%) were male. Further characteristics of the study population at baseline are shown in Table 1.

### Distribution of SELENOP

The mean (SD) SELENOP concentration was 4.8 (1.4) mg/L at baseline and approximately normally distributed (Suppl. Figure 1). With 3.9 (1.0) mg/L, the mean (SD) SELENOP concentration was lower at the 5-year follow-up than at baseline.

### Determinants of SELENOP concentration

In a logistic regression model with all baseline characteristics of Table 1 as independent variables and the baseline bottom SELENOP tertile versus the middle and top SELENOP tertiles as the dependent variable, the following determinants of a low SELENOP concentration was identified with forward selection: older age, male sex, BMI (inverse), medium or high physical activity (inverse), no or very high alcohol consumption, daily multivitamin/-mineral supplements use (inverse), CVD, diabetes mellitus (inverse), inflammation, and vitamin D deficiency or insufficiency (Table 2). In the longitudinal analysis using the same methods, the bottom SELENOP tertile from the 5-year follow-up was used as the dependent variable. As expected, the baseline SELENOP concentration was the strongest determinant of the SELENOP concentration at the 5-year follow-up. From the determinants of a low SELENOP concentration in the cross-sectional analysis, age, CVD and vitamin D deficiency were also selected for the final model of the longitudinal analysis. Further factors, not previously selected in the cross-sectional analysis, were current smoking, history of cancer, and dyslipidemia (inverse).

**Table 2:** Cross-sectional and longitudinal associations of baseline characteristics with the bottom tertile of the selenoprotein P serum concentration

Characteristic	Cross-sectional analysis (n = 7186)	Longitudinal analysis (n = 4164)
	OR (95%CI) ^a^	OR (95%CI) ^a^
Age (per 10 years)	1.15 (1.06; 1.24)	1.26 (1.12; 1.41)
Sex (Male)	1.53 (1.37; 1.71)	-
BMI (kg/m²)		
< 25	Ref	-
25 - <30	0.79 (0.70; 0.89)	-
≥ 30	0.65 (0.57; 0.76)	-
Smoking behaviour		
Never smoker	-	Ref
Former smoker	-	Ref
Current smoker	-	1.41 (1.15; 1.73)
Physical activity		
Inactive	Ref	-
Low	Ref	-
Medium or high	0.81 (0.73; 0.91)	-
Alcohol consumption		
Abstainer	1.18 (1.05; 1.33)	-
Moderate	Ref	-
High	Ref	-
Very high	1.76 (1.17; 2.64)	-
Multivitamin/-mineral supplements (daily)	0.63 (0.54; 0.74)	-
Diseases/conditions		
Cancer	-	1.35 (1.02; 1.79)
CVD	1.31 (1.14; 1.50)	1.30 (1.08; 1.56)
Diabetes mellitus	0.57 (0.49; 0.67)	-
Dyslipidemia	-	0.82 (0.71; 0.94)
Inflammation ^b^	1.36 (1.22; 1.51)	-
Vitamin D status ^c^		
Sufficient	Ref	Ref
Insufficient	1.18 (1.05; 1.32)	Ref
Deficient	1.73 (1.50; 2.01)	1.39 (1.13; 1.70)
Baseline SELENOP (per 1 mg/L)	NA	0.55 (0.51; 0.58)

Abbreviations: BMI, body mass index; CVD, cardiovascular disease; NA, not applicable; OR (95%CI), odds ratio and 95% confidence interval; Ref, reference category; SELENOP, selenoprotein P

^a^ Result of a logistic regression model with forward selection. The model contains all variables listed in the column

^b^ C-reactive protein ≥ 3 mg/L

^c^ Vitamin D deficient, 25-hydroxyvitamin D (25(OH)D) < 30 nmol/L; insufficient, 25(OH)D 30 - < 50 nmol/L; sufficient, 25(OH)D ≥ 50 nmol/L

### Association of SELENOP concentration with mortality

During a median follow-up time of 17.3 years (min: 15.9, max: 17.9 years), 2,126 (29.6%) study participants died. The primary causes of deaths were allocated to CVD (n = 709), cancer (n = 696), respiratory diseases (n = 111), gastrointestinal diseases (n = 105), psychiatric/neurological diseases (n = 123), and other/unknown causes (n = 382).

**Fig. 2:**
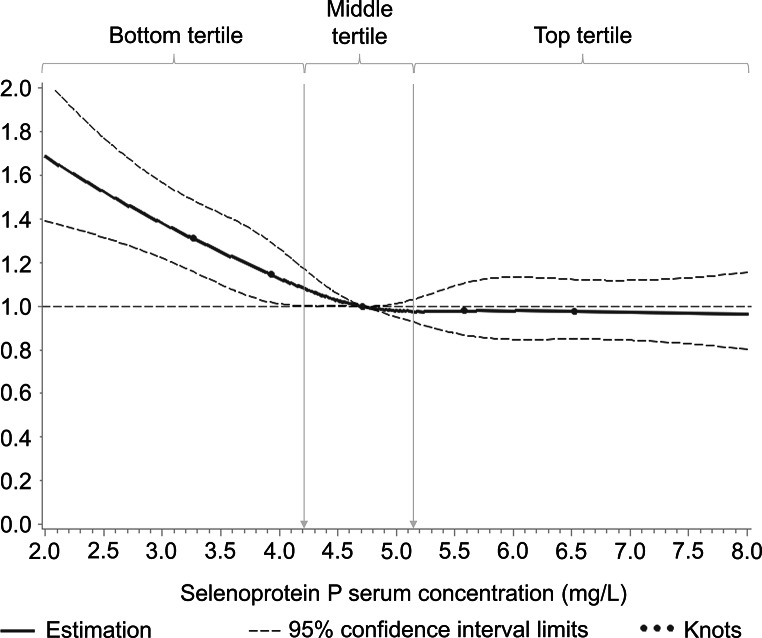
Concentration-response relationship between baseline serum selenoprotein P concentration and 17-year all-cause mortality Curves were assessed by restricted cubic splines with knots at the 10th, 25th, 50th, 75th and 90th selenoprotein P concentration percentile and the median as used as reference. As the SAS macro for concentration-response relationships does not work with multiple data sets, it was decided à priori to use imputed data set number 1 for this analysis

The concentration-response relationship of SELENOP concentrations with all-cause mortality was L-shaped (Fig. 2). Whereas no association was observed at SELENOP concentrations greater than the median, the restricted cubic splines curve showed increasing mortality with decreasing SELENOP concentrations below the median (4.7 mg/L), which reached statistical significance at SELENOP concentrations below 4.1 mg/L. This threshold for statistical significance is near the cut-off of the bottom tertile (4.2 mg/L).

Table 3 shows the associations of SELENOP tertiles with 17-year all-cause and cause-specific mortality using time-dependent modelling for the SELENOP measurements and all covariates except sex. Although effect estimates of the age- and sex adjusted model got slightly attenuated in the main model, the bottom SELENOP tertile (compared to the top tertile) remained statistically significantly associated with all mortality outcomes except mortality due to psychiatric/neurological diseases and other/unknown causes. The main model incorporated all identified determinants of a low SELENOP concentration (either in cross-sectional or longitudinal analyses, Table 2), which were also associated with all-cause mortality (which were all except multivitamin/-mineral use; Suppl. Table 1). The strengths of the associations in the main model were comparable for all-cause mortality, CVD mortality, and cancer mortality (around 1.3-fold increased mortality in the top tertile), whereas the associations with respiratory and gastrointestinal disease mortality were particularly strong (around 2-fold increase mortality). The middle SELENOP tertile was not associated with any outcome when compared to the top tertile.

**Table 3:** Associations of the selenoprotein P (SELENOP) serum concentration with all-cause and cause specific mortality during 17.3 years of follow-up

Outcome	SELENOP tertiles ^a^	n_total_	n_deaths_ (%)	Age and sex adjusted model	Main model ^b^
				HR (95%CI)	HR (95%CI)
All-cause mortality	T1	2395	857 (35.8)	**1.48 (1.33; 1.64)**	**1.35 (1.21; 1.50)**
	T2	2395	653 (27.3)	1.02 (0.91; 1.14)	1.02 (0.91; 1.14)
	T3	2396	616 (25.7)	Ref	Ref
CVD mortality ^c^	T1	2395	275 (11.5)	**1.33 (1.12; 1.59)**	**1.24 (1.04; 1.49)**
	T2	2395	217 (9.1)	0.85 (0.70; 1.04)	0.86 (0.71; 1.05)
	T3	2396	217 (9.1)	Ref	Ref
Cancer mortality ^d^	T1	2395	271 (11.3)	**1.47 (1.22; 1.77)**	**1.31 (1.09; 1.58)**
	T2	2395	225 (9.4)	1.11 (0.91; 1.35)	1.12 (0.92; 1.36)
	T3	2396	200 (8.4)	Ref	Ref
Respiratory disease mortality ^e^	T1	2395	61 (2.6)	**2.37 (1.49; 3.77)**	**2.06 (1.28; 3.32)**
	T2	2395	19 (0.8)	0.88 (0.50; 1.55)	0.87 (0.49; 1.53)
	T3	2396	31 (1.3)	Ref	Ref
Gastrointestinal disease mortality ^f^	T1	2395	54 (2.2)	**2.22 (1.37; 3.59)**	**2.04 (1.25; 3.32)**
	T2	2395	25 (1.0)	1.05 (0.60; 1.83)	1.03 (0.59; 1.80)
	T3	2396	26 (1.1)	Ref	Ref
Psychiatric/neurological disease mortality ^g^	T1	2395	48 (2.0)	1.47 (0.95; 2.29)	1.40 (0.89; 2.19)
	T2	2395	44 (1.8)	1.15 (0.72; 1.82)	1.15 (0.73; 1.84)
	T3	2396	31 (1.3)	Ref	Ref
Other/unknown cause of death ^h^	T1	2395	148 (6.2)	**1.34 (1.04; 1.72)**	1.27 (0.98; 1.63)
	T2	2395	123 (5.1)	1.16 (0.89; 1.49)	1.18 (0.91; 1.53)
	T3	2396	111 (4.6)	Ref	Ref

Abbreviations: CVD, cardiovascular disease; HR (95%CI), hazard ratio and 95% confidence interval; n_total_, sample size; n_deaths_ number of deaths; T1, T2, T3, bottom, middle and top tertile, respectively; Ref, Reference category; SELENOP, selenoprotein P

Printed in bold: Statistically significant (*P* < 0.05)

^a^ Cut-offs for the SELENOP concentration tertiles at baseline: Tertile 1: < 4.21 mg/L; Tertile 3: > 5.25 mg/L. Cut-offs for the SELENOP concentration tertiles at 5-year follow-up: Tertile 1: < 3.54 mg/L; Tertile 3: > 4.26 mg/L

^b^ Adjusted for age, sex, BMI, current smoking, physical activity, alcohol consumption, history of cancer, cardiovascular disease, diabetes mellitus, dyslipidaemia, inflammation (C-reactive protein ≥ 3 mg/l), and vitamin D status. SELENOP concentration tertiles and all covariates except sex were modelled as time-dependent variables by considering their values at the 5-year follow-up of the ESTHER study

^c^ ICD 10 codes I00-I99

^d^ ICD 10 codes C00-C97

^e^ ICD 10 codes J00-J99

^f^ ICD 10 codes K00-K93

^g^ ICD 10 codes F00-G99

^h^ ICD 10 codes A00-B99, D50-D90, E00-E90, M00-M99, N00-N40, R00-R99, S00-T98

Slightly weaker effect estimates were observed for most outcomes when only the baseline SELENOP concentration and covariate values were used and the variables were not modelled as time-dependent variables (Suppl. Table 2). If deaths, which occurred earlier (year 1–9) or later (year 10–18) during follow-up, were analysed distinctly, the effect estimate for all-cause mortality was the same in both analyses (Suppl. Table 3). However, there were differences for cause-specific mortality. The bottom SELENOP tertile was only associated with cancer deaths that occurred later in follow-up. In contrast, the associations of the SELENOP concentration with CVD mortality and with deaths neither caused by CVD nor cancer were stronger with deaths that occurred earlier in the follow-up but an association was still detectable with later deaths (albeit not statistical significantly for CVD mortality).

In subgroup analyses, a stronger association of the bottom SELENOP tertile with all-cause mortality and cancer mortality was observed among men than among women, whereas the effect estimates for CVD mortality were comparable (Table 4). In tests for potential interactions of the SELENOP concentration with the variables of the main model for the outcome all-cause mortality, the interaction term of the bottom SELENOP tertile (versus the top tertile) and sex was the only statistically significant one (*p* = 0.008, Suppl. Table 4).

**Table 4:** Subgroup analyses for the association of bottom versus top tertile of the selenoprotein P serum concentration with all-cause and cause-specific mortality during 17.3 years of follow-up

Subgroup	n_total_	All-cause mortality		CVD mortality		Cancer mortality	
		n_deaths_ (%)	HR^a^ (95%CI)	n_deaths_ (%)	HR^a^ (95%CI)	n_deaths_ (%)	HR^a^ (95%CI)
Total population	7186	2126 (29.6)	**1.35 (1.21; 1.50)**	709 (9.9)	**1.24 (1.04; 1.49)**	696 (9.7)	**1.31 (1.09; 1.58)**
Women	3967	912 (23.0)	**1.20 (1.03; 1.42)**	303 (7.6)	1.22 (0.92; 1.61)	284 (7.2)	1.16 (0.86; 1.55)
Men	3219	1214 (37.7)	**1.47 (1.28; 1.70)**	406 (12.6)	**1.27 (1.01; 1.62)**	412 (12.8)	**1.42 (1.11; 1.82)**
< 65 years	4324	802 (18.6)	**1.48 (1.24; 1.75)**	210 (4.9)	**1.45 (1.04; 2.04)**	336 (7.8)	1.25 (0.95; 1.64)
≥ 65 years	2862	1324 (46.3)	**1.26 (1.10; 1.44)**	499 (17.4)	1.15 (0.93; 1.42)	360 (12.6)	**1.33 (1.03; 1.73)**
No CVD	6010	1525 (25.4)	**1.30 (1.15; 1.47)**	459 (7.6)	1.20 (0.95; 1.49)	548 (9.1)	**1.26 (1.02; 1.55)**
CVD	1176	601 (51.1)	**1.52 (1.24; 1.86)**	250 (21.3)	1.33 (0.98; 1.80)	148 (12.6)	**1.58 (1.02; 2.45)**
No history of cancer	6728	1897 (28.2)	**1.36 (1.21; 1.52)**	652 (9.7)	**1.20 (1.00; 1.45)**	575 (8.6)	**1.32 (1.08; 1.63)**
History of cancer	458	229 (50.0)	1.26 (0.91; 1.73)	57 (12.5)	1.60 (0.77; 3.32)	121 (26.4)	1.21 (0.78; 1.88)

Abbreviations: CVD, cardiovascular disease; HR (95%CI), hazard ratio and 95% confidence interval; n_total_, sample size; n_deaths_ number of deaths; T1, T2, T3, bottom, middle and top tertile, respectively; Ref, Reference category; SELENOP, selenoprotein P

Printed in bold: Statistically significant (*P* < 0.05)

^a^ Adjusted for age, sex, BMI, current smoking, physical activity, alcohol consumption, history of cancer, cardiovascular disease, diabetes mellitus, dyslipidaemia, inflammation (C-reactive protein ≥ 3 mg/l), and vitamin D status. SELENOP concentration tertiles and all covariates except sex were modelled as time-dependent variables by considering their values at the 5-year follow-up of the ESTHER study

Although no interactions of the SELENOP concentration with age, CVD and history of cancer, were observed, there were some differences in the results according to these factors. Effect estimates for all-cause mortality and CVD mortality were stronger among subjects younger than 65 years compared to older subjects. Furthermore, effect estimates for all mortality outcomes were stronger among subjects with CVD at baseline. Lastly, the effect estimate for cancer mortality was stronger among subjects without a history of cancer compared to subjects with a history of cancer.

## Discussion

### Summary

In this large population-based cohort study with repeated measurements, the serum SELENOP concentration was strongly and statistically significantly associated with all-cause mortality and mortality due to CVD, cancer, respiratory diseases, and gastrointestinal diseases. The association of SELENOP concentrations with all-cause mortality was found to be a non-linear inverse association with mortality starting to increase significantly at SELENOP concentrations below 4.1 mg/L. For subjects with SELENOP concentrations < 4.2 mg/L, mortality was increased by 35%. The excess mortality was more than twice as high for men compared to women (mortality increased by 47% vs. 20%) and a statistically significant interaction of the SELENOP concentration with sex was determined.

### General comparison with previous studies & novelty

The mean baseline SELENOP concentration in the ESTHER study (4.8 mg/L) was comparable with the one from the German Modulation of Cardiovascular Risk Factors (MoKaRi) study (5.0 mg/L), a cross-sectional study of 51 participants with a mean age (61 years) comparable with the one of the ESTHER study (62 years) [37].

To the best of our knowledge, ESTHER is the second population-based cohort study to assess the association of SELENOP concentrations with mortality as all previous studies, except the Malmö Preventive Project (MPP) [38], measured serum selenium to determine the selenium status. However, our results are partly comparable with these studies because SELENOP accounts for more than 50% of circulating selenium [2, 3] and serum selenium and SELENOP concentration were found to be highly correlated in our study (r = 0.69) and other studies (r = 0.60–0.78 [38–41]). Novel aspects in our study are the repeated SELENOP measurements during follow-up and more cause-specific mortality endpoints than CVD and cancer mortality.

### Age and SELENOP

Each 10 years of advancing age were associated with a 1.15 and 1.26-fold increased odds of having a low SELENOP concentration (categorized in the bottom SELENOP tertile) in the cross-sectional and longitudinal analysis, respectively. Results of previous cross-sectional studies were mixed and there are reports of no association of biomarkers of selenium status with age [41, 42] as well as reports of positive [43, 44] and inverse associations with age [45]. The mixed results could be explained by different age-ranges in the studies, sex-specific differences, and the fact that a large sample size is needed to detect a weak risk factor with an odds ratio of approx. 1.2.

### Association of selenium status with mortality

The Swedish MPP is the only population-based cohort study, which also assessed the association of SELENOP concentrations and mortality [38]. In agreement with our results, the lowest SELENOP quintile was strongly associated with all-cause mortality (HR [95%CI]: 1.51 [1.32; 1.72]) and CVD mortality (HR [95%CI]: 1.61 [1.32; 2.09]) when compared to SELENOP quintiles 2–5. Other cause-specific mortality outcomes were not analyzed.

A systematic PubMed search was conducted at Dec 6th, 2023 using the key words “selenium [title/abstract]” and “mortality [title/abstract]” to identify other population-based cohort studies, which reported results on the association of measured serum/plasma selenium and any mortality outcome and the detailed results are shown in Suppl. Table 5. After removing 3 duplicate publications from the same cohort [46–48], 21 studies were identified. Regarding all-cause mortality, 5 studies reported a non-significant [42, 49–52] and the majority of 11 studies a statistical significant [22, 23, 53–61] inverse association of the serum/plasma selenium concentration with all-cause mortality. Regarding CVD mortality, 5 studies reported non-significant [22, 50, 54, 57, 62] and 5 studies significant findings [23, 58, 60, 61, 63]. For cancer mortality, 4 studies reported non-significant [22, 57, 58, 64] and 3 studies significant findings [54,65, 66]. Differences in statistical power are the most likely explanation for the non-significant results because all results pointed in the same direction. For other causes of mortality, there was only one report on respiratory disease mortality from the British National Diet and Nutrition Survey [57]. In agreement with our study, a strong, inverse association of the serum selenium concentration with the aforementioned outcome was observed in an age- and sex-adjusted model (HR [95%CI] per 1 SD increase: 0.65 [0.51; 0.82]) but it lost its statistical significance in a more comprehensively adjusted model (OR not reported).

An analysis of the Dutch Prevention of Renal and Vascular End-Stage Disease (PREVEND) study [42] and two analyses with different cohorts of the US-American NHANES [47, 48] also conducted concentration-response analyses and, consistent with our results, found a non-linear association of selenium concentrations with all-cause mortality. The three studies also observed a strong inverse association of selenium concentrations and mortality in the bottom tertile whereas there was no association in the middle tertile of the selenium distribution (a so-called L-shaped association). The association of a high selenium status with mortality was inconsistent. Whereas there was no increased mortality in subjects with a high selenium status in our study and the NHANES from 2011 to 2016 [48], a modestly decreased mortality was suggested in the PREVEND study [42], and a modestly increased mortality was suggested in the NHANES from 1988 to 1994 [47]. Of note, none of the deviations from “no association” reached statistical significance at higher selenium concentrations.

### Interaction with sex

We detected a statistically significant interaction of the SELENOP concentration with sex for the outcome all-cause mortality in our study. Interestingly, this interaction was also detected in the PREVEND study for all-cause mortality and in the Belgian Interuniversity Study on Nutrition and Health (BIRNH) for cancer mortality, and both studies also observed a stronger association of a low selenium status with (cancer) mortality in men than in women [42, 65]. In contrast, no sex difference was observed in the US-American NHANES [22, 47] and a Chinese study [50], while all other comparable studies did not report results stratified by sex. Maybe, this interaction with sex is specific for European countries with low-to-moderate selenium supply as it was now detected in studies from Belgium, the Netherlands and Germany. We may only speculate on the reasons for the observed sex difference. Presumably sex-specific differences in the hierarchical regulation of selenoprotein expression play a role [9]. When selenium was injected into selenium deficient rats, females tended to retain the trace element more efficiently than males in most organs with the exception of the gonads [67]. The retention of selenium in these organs might be of particular importance to protect them against cancer because we observed the sex difference for cancer mortality and not for CVD mortality in our study. Strikingly, sex-specific meta-analyses for the associations of high selenium status and cancer risk pointed also in the direction of a sex difference [68]. The ORs [95%CIs] were 0.74 (0.64; 0.86) and 0.90 (0.86; 0.95) among men and women, respectively, and overlapped just minimally. Selenium has been ascribed anti-cancerogenic effects by anti-oxidative effects and effects on DNA stability [69].

### Analyses addressing potential reverse causality

Reverse causality in this context would be present if low SELENOP concentrations are caused by diseases and not the other way around and that the observed increased mortality of subjects with low SELENOP concentrations is caused by the diseases whereas the low SELENOP concentration is an uninvolved bystander. Two sensitivity analyses were carried out to address potential reverse causality. First, we excluded subjects with a history of CVD or cancer from the analyses (Table 4). For all-cause mortality, it did not change much. Results for CVD mortality were slightly weaker (HR [95%CI]: 1.20 (0.95; 1.49)) in subjects without a history of CVD than among subjects with CVD (HR [95%CI]: 1.33 (0.98; 1.80)). Albeit not statistically significant, both point estimates are quite similar. For cancer mortality, it was the opposite, with a slightly stronger results in subjects without a history of cancer at baseline (HR [95%CI]: 1.32 (1.08; 1.63)) compared to study participants with a history of cancer (HR [95%CI]: 1.21 (0.78; 1.88)). The bottom SELENOP tertile was only associated with cancer deaths that occurred later in follow-up. Second, we excluded events that happened in the first 9 years of follow-up, which could have been caused by already existing (maybe undiagnosed) diseases at baseline (Suppl. Table 3). Again here, results for all-cause mortality were unaltered and results for cancer mortality were stronger than in the main analysis. In contrast, and similarly to the first analysis addressing reverse causality, the associations of the SELENOP concentration with CVD mortality were weaker and not statistically significant (1.12 (0.89; 1.41)). Thus, the two analyses addressing reverse causality come to the same conclusion. Whereas the results raised no concerns towards potential bias by reverse causality for all-cause mortality and cancer mortality, it cannot be excluded as an explanation for the observed association of SELONOP concentrations and CVD mortality.

### Strengths and limitations

Strengths of this study include the time-dependent modelling of SELENOP concentrations and covariates, the adjustment for a wide range of potential confounders, an almost complete registry-based follow-up, and the use of a calibrated SELENOP assay. Furthermore, the large size of the cohort and a large number of deaths identified via 17 years of follow-up enabled us to obtain precise effect estimates and address more causes of death than just CVD and cancer.

The main limitation of our prospective cohort study is its observational nature. Despite adjustment for known potential confounders, we cannot rule out that a low serum SELENOP concentration is only a non-specific marker for a poor health status, which is confounded by other unconsidered factors.

Another limitation is that the SRM1950 used to standardize our SELENOP assay was developed in an US-American sample with 77% of the study participants being of Caucasian ethnicity while relatively large proportions were of African American or Hispanic ethnicity. The ESTHER study participants are almost all of Caucasian ethnicity. However, this limitation only affects absolute SELENOP values. The relative comparisons of SELENOP tertiles for mortality outcomes are not affected by the chosen SRM and the reported HRs and 95%CIs would have been exactly the same with a SRM specifically developed for the European population. As, up to date, no such SRM is available, there is still a need to develop it and to conduct a survey with it to establish reference values for SELENOP concentrations among the European population.

### Public health implications

A clinical trial showed that sodium selenit can effectively increase the serum SELENOP concentration alongside the serum selenium concentration [39]. Meta-analyses of observational studies observed statistical significant associations of a low serum/plasma selenium concentration with increased all-cause, CVD and cancer mortality [70–72]. However, meta-analyses of randomized controlled trials (RCTs) did not detect an effect of selenium supplementation on these mortality outcomes [72, 73]. The majority of the weight in these meta-analyses is attributed to the Selenium and Vitamin E Cancer Prevention Trial (SELECT) trial [74]. This large trial has a major limitation that may explain the null results. It was conducted in the US, a country with high selenium status, and the median baseline serum selenium concentration was 135 µg/L. This serum concentration markedly exceeds the threshold of 90 µg/L, which is already sufficient to saturate the anti-oxidative GPX activity at its maximum [75]. Thus, by not taking into account the L-shaped association of the selenium concentration and mortality, the majority of the study population could not profit from the intervention.

In addition, there are many Mendelian randomization (MR) studies to date for selenium and various diseases. Most did not find a relationship including one between serum selenium concentrations and longevity [76]. However, there is also a growing number of MR studies, that establish relationships between genetically determined selenium concentrations and diseases (e.g., for colorectal cancer, nonalcoholic fatty liver disease, type 2 diabetes, chronic kidney disease, ulcerative colitis, and schizophrenia [77–82]). The same problem of not considering the L-shaped association of selenium concentrations with mortality also affects MR studies. A lesson can be learned from 25(OH)D levels, which also have an L-shaped association with mortality. In contrast to several other MR studies, which used the whole range of 25(OH)D levels and observed no relationship, a new one using only subjects with low 25(OH)D levels detected a causal relationship between 25(OH)D levels and all-cause mortality [83]. New trials and MR studies are needed that include only subjects with a low selenium status.

## Conclusions

To date, our study is the second largest population-based cohort study on the association of selenium status and mortality worldwide after the US-American NHANES [47] and thus the largest study from Europe so far, where selenium supply is insufficient. The bottom SELENOP tertile was strongly associated with all-cause, CVD, cancer, respiratory disease and gastrointestinal disease mortality. The association of SELENOP concentrations with all-cause mortality was found to be a non-linear inverse association with risk starting to increase at SELENOP concentrations < 4.1 mg/L. Associations were stronger among men than women.

The associations of the SELENOP concentration with multiple major mortality outcomes might be explained by its essential role in selenium transport and hence its indirect anti-oxidative effects. The observed L-shaped association of SELENOP concentrations and all-cause mortality study implies that new trials testing the efficacy of selenium supplementation in subjects with a low SELENOP concentration would be promising despite the null results from previous trials, which did not include a sufficient fraction of study participants with a low selenium status.

### Electronic supplementary material

Below is the link to the electronic supplementary material.

Supplementary Material 1

## Data Availability

No data will be shared because the German Cancer Research Center is the owner of the data of the ESTHER study and does not provide open data access for this cohort study.

## List of Abbreviations

95%CI: 95% confidence interval
BIRNH: Belgian Interuniversity Study on Nutrition and Health
COV: Coefficient of variation
CVD: Cardiovascular disease
eGFR: Estimated glomerular filtration rate
ESTHER: Epidemiologische Studie zu Chancen der Verhütung, Früherkennung und optimierten Therapie chronischer Erkrankungen in der älteren Bevölkerung
GPs: General practitioners
GPX: Glutathione peroxidase
HR: Hazard ratio
IOM: Institute of Medicine
NHANES: National Health and Nutrition Examination Survey
MPP: Malmö Preventive Project
MR: Mendelian Randomization
OR: Odds ratio
PREVEND: Prevention of Renal and Vascular End-Stage Disease
RCTs: Randomized controlled trials
SELECT: Selenium and Vitamin E Cancer Prevention Trial
SELENOP: Selenoprotein P
