# Supplementary material for: Strong associations of serum selenoprotein P with all-cause mortality and mortality due to cancer, cardiovascular, respiratory and gastrointestinal diseases in older German adults
Source: Eur J Epidemiol. 2024 Jan 10;39(2):121–36. doi: 10.1007/s10654-023-01091-4 (PMC10904445; doi:10.1007/s10654-023-01091-4)
Supplement: Supplementary file 1 — Supplementary Material 1 [file 10654_2023_1091_MOESM1_ESM.docx]

**Supplemental Material to**

**Strong associations of serum selenoprotein P with all-cause mortality and mortality due to cancer, cardiovascular, respiratory and gastrointestinal diseases in a large cohort of older adults from Germany**

**Table of Contents**

[**Suppl. Figure 1** – Histogram of baseline selenoprotein P serum concentrations measured at baseline 2](#_Toc124418810)

[**Suppl. Table 1** – Test whether baseline characteristics associated with a low selenoprotein P concentration are also associated with all-cause mortality 3](#_Toc124418812)

[**Suppl. Table 2** – Sensitivity analysis on mortality outcomes without time-dependent modelling of selenoprotein P concentrations and covariates using only the baseline values 4](#_Toc124418813)

[**Suppl. Table 3** – Associations of baseline selenoprotein P serum concentrations with earlier (year 1-9) and later (year 10-18) deaths during follow-up](#_Toc124418814) 6

[**Suppl. Table 4** – Tests for interactions of the bottom selenoprotein P tertile and variables of the main model with respect to the outcome all-cause mortality](#_Toc124418815) 7

**Suppl. Table 5 –** Associations of serum or plasma selenium concentrations with mortality outcomes in

population-based cohort studies…………………………………………………………………………………….8

**Suppl. Figure 1** – Histogram of selenoprotein P serum concentrations measured at baseline


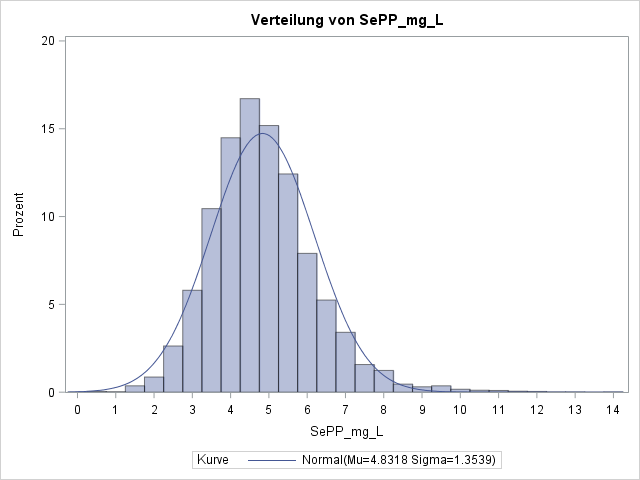


Percent

Selenoprotein P concentration (mg/L)

Note: The curve shows a perfect normal distribution estimated from the data

#

# **Suppl. Table 1** – Test whether baseline characteristics associated with a low selenoprotein P concentration are also associated with all-cause mortality

| **Characteristic** | **HR (95%CI) ^a^** |
| --- | --- |
| Age (per 10 years) | **2.85 (2.64; 3.08)** |
| Sex (Male) | **1.98 (1.80; 2.19)** |
| BMI (kg/m²) |  |
| < 25 | Ref |
| 25 - <30 | 0.89 (0.80; 0.99) |
| ≥ 30 | **1.14 (1.01; 1.29)** |
| Current smoker | **2.12 (1.90; 2.37)** |
| Medium or high  physical activity | 0.75 (0.68; 0.84) |
| Alcohol consumption |  |
| Abstainer | **1.15 (1.03; 1.30)** |
| Moderate or high | Ref |
| Very high | 1.25 (0.90; 1.74) |
| Multivitamin/mineral  supplements (daily) | 0.97 (0.86; 1.11) |
| Diseases/conditions |  |
| Cancer | **1.88 (1.64; 2.16)** |
| CVD | **1.57 (1.43; 1.74)** |
| Diabetes mellitus | **1.58 (1.42; 1.75)** |
| Dyslipidaemia | **0.90 (0.82; 0.98)** |
| Inflammation ^b^ | **1.33 (1.22; 1.45)** |
| Vitamin D status ^c^ |  |
| Sufficient | Ref |
| Insufficient | 1.09 (0.99; 1.21) |
| Deficient | **1.42 (1.25; 1.61)** |

Abbreviations: BMI, body mass index; CVD, cardiovascular disease; CRP, C-reactive protein; HR (95% CI), hazard ratio and 95% confidence interval; Ref, reference category.

^a^ Adjusted for all variables in the table.

^b^ C-reactive protein ≥ 3 mg/L

^c^ Vitamin D deficient, 25-hydroxyvitamin D (25(OH)D) < 30 nmol/L; insufficient, 25(OH)D 30 - < 50 nmol/L; sufficient, 25(OH)D ≥ 50 nmol/L.

# **Suppl. Table 2** – Sensitivity analysis on mortality outcomes without time-dependent modelling of selenoprotein P concentrations and covariates using only the baseline values

| Outcome | SELENOP ^a^ | n_total_ | n_deaths_ (%) | Age and sex  adjusted model | Main model ^b^ |
| --- | --- | --- | --- | --- | --- |
|  |  |  |  | HR (95%CI) | HR (95%CI) |
| All-cause | T1 | 2395 | 857 (35.8) | **1.35 (1.21; 1.49)** | **1.29 (1.16; 1.44)** |
| mortality | T2 | 2395 | 653 (27.3) | 1.03 (0.92; 1.15) | 1.03 (0.92; 1.15) |
|  | T3 | 2396 | 616 (25.7) | Ref | Ref |
|  | Per 1 SD | 7186 | 2126 (29.6) | **0.86 (0.82; 0.90)** | **0.89 (0.85; 0.93)** |
|  |  |  |  |  |  |
| CVD ^c^ | T1 | 2395 | 275 (11.5) | **1.20 (1.01; 1.44)** | 1.18 (0.98; 1.42) |
| mortality | T2 | 2395 | 217 (9.1) | 0.96 (0.79; 1.16) | 0.97 (0.80; 1.17) |
|  | T3 | 2396 | 217 (9.1) | Ref | Ref |
|  | Per 1 SD | 7186 | 2126 (29.6) | **0.92 (0.85; 0.99)** | 0.93 (0.86; 1.01) |
|  |  |  |  |  |  |
| Cancer ^d^ | T1 | 2395 | 271 (11.3) | **1.32 (1.10; 1.59)** | **1.24 (1.03; 1.50)** |
| mortality | T2 | 2395 | 225 (9.4) | 1.09 (0.90; 1.32) | 1.07 (0.88; 1.30) |
|  | T3 | 2396 | 200 (8.4) | Ref | Ref |
|  | Per 1 SD | 7186 | 2126 (29.6) | **0.87 (0.80; 0.94)** | **0.90 (0.83; 0.97)** |
|  |  |  |  |  |  |
| Respiratory | T1 | 2395 | 61 (2.6) | **1.86 (1.20; 2.87)** | **1.63 (1.03; 2.56)** |
| disease | T2 | 2395 | 19 (0.8) | 0.58 (0.33; 1.03) | 0.58 (0.32; 1.02) |
| mortality ^e^ | T3 | 2396 | 31 (1.3) | Ref | Ref |
|  | Per 1 SD | 7186 | 2126 (29.6) | **0.64 (0.52; 0.79)** | **0.70 (0.56; 0.87)** |
|  |  |  |  |  |  |
| Gastrointestinal | T1 | 2395 | 54 (2.2) | **2.06 (1.29; 3.29)** | **1.89 (1.17; 3.07)** |
| disease | T2 | 2395 | 25 (1.0) | 0.94 (0.54; 1.63) | 0.95 (0.55; 1.65) |
| mortality ^f^ | T3 | 2396 | 26 (1.1) | Ref | Ref |
|  | Per 1 SD | 7186 | 2126 (29.6) | **0.72 (0.58; 0.89)** | **0.75 (0.60; 0.94)** |
|  |  |  |  |  |  |
| Psychiatric | T1 | 2395 | 48 (2.0) | **1.58 (1.00; 2.48)** | 1.56 (0.98; 2.48) |
| disease | T2 | 2395 | 44 (1.8) | 1.39 (0.88; 2.20) | 1.43 (0.90; 2.27) |
| mortality ^g^ | T3 | 2396 | 31 (1.3) | Ref | Ref |
|  | Per 1 SD | 7186 | 2126 (29.6) | **0.81 (0.67; 0.98)** | 0.82 (0.68; 1.00) |
|  |  |  |  |  |  |
| Other/unknown | T1 | 2395 | 148 (6.2) | **1.31 (1.02; 1.67)** | **1.32 (1.03; 1.71)** |
| cause of death ^h^ | T2 | 2395 | 123 (5.1) | 1.08 (0.83; 1.39) | 1.11 (0.86; 1.44) |
|  | T3 | 2396 | 111 (4.6) | Ref | Ref |
|  | Per 1 SD | 7186 | 2126 (29.6) | **0.88 (0.79; 0.98)** | **0.89 (0.80; 0.99)** |

Abbreviations: CVD, cardiovascular disease; HR (95% CI), hazard ratio and 95% confidence interval; n_total_, sample size; n_deaths,_ number of deaths; T1, T2, T3, bottom, middle and top tertile, respectively; Ref, Reference category; SD, standard deviation; SELENOP, selenoprotein P.

Printed in bold: Statistically significant (*P* < 0.05).

^a^ Cut-offs for SELENOP concentration tertiles at baseline: Tertile 1: < 4.21 mg/L; Tertile 3: > 5.25 mg/L. SD=1.354 mg/L.

^b^ Adjusted for age, sex, BMI, current smoking, physical activity, alcohol consumption, history of cancer, cardiovascular disease, diabetes mellitus, dyslipidaemia, inflammation (C-reactive protein ≥ 3 mg/l), and vitamin D status.

^c^ ICD 10 codes I00-I99; ^d^ ICD 10 codes C00-C97; ^e^ ICD 10 codes J00-J99; ^f^ ICD 10 codes K00-K93; ^g^ ICD 10 codes F00-G99; ^h^ ICD 10 codes A00-B99, D50-D90, E00-E90, M00-M99, N00-N40, R00-R99, S00-T98

# **Suppl. Table 3** – Associations of the baseline selenoprotein P serum concentration with earlier (year 1-9) and later (year 10-18) deaths during follow-up

| Outcome | SELENOP  tertiles ^a^ | Association with deaths in year 1-9 of follow-up | | |  | Association with deaths in year 10-18 of follow-up | | |
| --- | --- | --- | --- | --- | --- | --- | --- | --- |
|  |  | n_total_ | n_deaths_ (%) | HR (95%CI) ^b^ |  | n_total_ ^c^ | n_deaths_ (%) | HR (95%CI) ^b^ |
| All-cause | T1 | 2395 | 332 (13.9) | **1.29 (1.08; 1.54)** |  | 2063 | 525 (25.5) | **1.29 (1.13; 1.48)** |
| mortality | T2 | 2395 | 195 (8.1) | 0.84 (0.69; 1.02) |  | 2200 | 458 (20.8) | 1.13 (0.99; 1.30) |
|  | T3 | 2396 | 222 (9.3) | Ref |  | 2174 | 394 (18.2) | Ref |
|  |  |  |  |  |  |  |  |  |
| CVD | T1 | 2395 | 106 (4.4) | 1.28 (0.94; 1.74) |  | 2063 | 169 (8.2) | 1.12 (0.89; 1.41) |
| mortality | T2 | 2395 | 56 (2.3) | 0.73 (0.52; 1.04) |  | 2200 | 161 (7.3) | 1.08 (0.87; 1.37) |
|  | T3 | 2396 | 75 (3.1) | Ref |  | 2174 | 142 (6.5) | Ref |
|  |  |  |  |  |  |  |  |  |
| Cancer | T1 | 2395 | 120 (5.0) | 1.05 (0.80; 1.39) |  | 2063 | 151 (7.3) | **1.41 (1.10; 1.83)** |
| mortality | T2 | 2395 | 93 (3.9) | 0.98 (0.91; 1.22) |  | 2200 | 132 (6.0) | 1.22 (0.94; 1.58) |
|  | T3 | 2396 | 95 (4.0) | Ref |  | 2174 | 105 (4.8) | Ref |
|  |  |  |  |  |  |  |  |  |
| Non-CVD, | T1 | 2395 | 106 (4.4) | **1.79 (1.27; 2.53)** |  | 2063 | 205 (9.9) | **1.39 (1.11; 1.73)** |
| non-cancer | T2 | 2395 | 46 (1.9) | 0.88 (0.59; 1.31) |  | 2200 | 165 (7.5) | 1.12 (0.89; 1.40) |
| mortality | T3 | 2396 | 52 (2.2) | Ref |  | 2174 | 147 (6.8) | Ref |

Abbreviations: CVD, cardiovascular disease; HR (95% CI), hazard ratio and 95% confidence interval; n_total_, sample size; n_deaths_, number of deaths; T1, T2, T3, bottom, middle and top tertile, respectively; Ref, Reference category; SELENOP, selenoprotein P.

Printed in bold: Statistically significant (*P* < 0.05).

^a^ Cut-offs for SELENOP concentration tertiles at baseline: Tertile 1: < 4.21 mg/L; Tertile 3: > 5.25 mg/L.

^b^ Adjusted for age, sex, BMI, current smoking, physical activity, alcohol consumption, history of cancer, cardiovascular disease, diabetes mellitus, dyslipidaemia, inflammation (C-reactive protein ≥ 3 mg/l), and vitamin D status.

^c^ Subjects who died in the first 9 years of follow-up were excluded.

# **Suppl. Table 4** – Tests for interactions of the bottom selenoprotein P tertile and variables of the main model with respect to the outcome all-cause mortality

| **Characteristic** | **ß coefficient of  product term ^a^** | **p-value** |
| --- | --- | --- |
| Age (per 10 years) | -0.004 | 0.556 |
| Sex (Male) | **0.237** | **0.008** |
| BMI (kg/m²) |  |  |
| < 25 | Ref |  |
| 25 - <30 | -0.083 | 0.442 |
| ≥ 30 | -0.187 | 0.115 |
| Current smoker | -0.112 | 0.311 |
| Medium or high  physical activity | 0.116 | 0.253 |
| Alcohol consumption |  |  |
| Abstainer | -0.037 | 0.722 |
| Moderate or high | Ref |  |
| Very high | 0.452 | 0.286 |
| Diseases/conditions |  |  |
| Cancer | 0.039 | 0.756 |
| CVD | 0.116 | 0.217 |
| Diabetes mellitus | 0.009 | 0.928 |
| Dyslipidaemia | 0.092 | 0.299 |
| Inflammation ^b^ | 0.042 | 0.671 |
| Vitamin D status ^c^ |  |  |
| Sufficient | Ref |  |
| Insufficient | <-0.001 | 0.997 |
| Deficient | -0.026 | 0.799 |

Abbreviations: BMI, body mass index; CVD, cardiovascular disease; CRP, C-reactive protein; Ref, reference category.

Printed in bold: P < 0.05.

^a^ Adjusted for age, sex, BMI, current smoking, physical activity, alcohol consumption, history of cancer, cardiovascular disease, diabetes mellitus, dyslipidaemia, inflammation (C-reactive protein ≥ 3 mg/l), and vitamin D status. SELENOP concentration and all covariates except sex were modelled as time-dependent variables by considering their values at the 5-year follow-up of the ESTHER study.

^b^ C-reactive protein ≥ 3 mg/L

^c^ Vitamin D deficient, 25-hydroxyvitamin D (25(OH)D) < 30 nmol/L; insufficient, 25(OH)D 30 - < 50 nmol/L; sufficient, 25(OH)D ≥ 50 nmol/L.

**Suppl. Table 5.** Associations of serum or plasma selenium concentrations with mortality outcomes in population-based cohort studies

| **First author,  publication year,**  **study name** | **Study**  **Acronym** | **Country** | **Age** ± **SD**  **(years)** | **FUP time**  **(years)** | **N_total_** | **N_deaths_^a^** | **Comparison**  **Selenium** | **All-cause**  **mortality** | **CVD mortality** | **Cancer mortality** | **Respiratory**  **disease mortality** |
| --- | --- | --- | --- | --- | --- | --- | --- | --- | --- | --- | --- |
|  |  |  |  |  |  |  |  | **RR (95%CI)** | **RR (95%CI)** | **RR (95%CI)** | **RR (95%CI)** |
| Salonen 1982 | - | Finland | 50.4 ± 6.3 | 7.0 | 414 | 131 | Low vs. high | - | **2.2 (1.2-4.0)** | - | - |
| Virtamo 1985 | - | Finland | 55-74 | 5.0 | 1,110 | 215 | Low vs. high | **1.4 (1.0-2.0)** | **1.6 (1.1-2.3)** | - | - |
| Kok 1987 | EPOZ | Netherlands | 67.9 ± 11.8^b^ | 9.0 | 252 | 84 | Low vs. high | - | 2.0 (0.8-5.0) | - | - |
| Kok 1987 | EPOZ | Netherlands | 60.2 ± 13.4^b^ | 9.0 | 207 | 69 | Low vs. high | - | - | **1.9 (1.0-3.5)** | - |
| Marniemi 1998 | - | Finland | ≥ 65 | 13 | 344 | 225 | Low vs. high | 1.11 (0.78-1.59) | - | - | - |
| Criqui 1991 | LRC | N. America | ≥ 30 | 8.5 | 374 | 136 | Per µg/dl incr. | - | - | p=0.43, RR n. r. | - |
| Kilander 2001 | - | Sweden | 48.6-51.1 | 25.7 | 2,285 | 630 | Per SD incr. | **0.87 (0.80-0.95)** | - | - | - |
| Wei 2004 | - | China | 56.6 ± 8.0 | 15.0 | 1,103 | 516 | Low vs. high | 1.08 (0.83-1.41) | 1.52 (0.95-2.43) | - | - |
| Kornitzer 2004 | BIRNH | Belgium | 25-74 | 10.0 | 804 | 201 | Low vs. high | - | - | **1.60 (1.02-2.49)^c^** | - |
| Akbaraly 2005 | EVA | France | 65 ± 3 | 9.0 | 1,389 | 101 | Low vs. high | **3.34 (1.71-6.53)** | p>0.05, RR n. r. | **1.79 (1.32-2.44)** | - |
| Walston 2006 | WHAS I | USA | ≥ 65 | 5.0 | 619 | 197 | Low vs. high | **1.54 (1.03-2.32)** | ~~-~~ | ~~-~~ | ~~-~~ |
| González 2007 | - | Spain | 75.1 ± 6.5 | 4.3 | 215 | 60 | Low vs. high | 1.77 (0.71-4.39) | ~~-~~ | ~~-~~ | ~~-~~ |
| Lauretani 2008 | InCHIANTI | Italy | 75.6 ± 7.4 | 6.0 | 1,042 | 237 | Low vs. high | **1.62 (1.06-2.43)** | - | - | - |
| Bates 2011 | BNDNS | UK | 76.6 ± 7.4 | 14.0 | 1,054 | 717 | Per SD incr. | **0.83 (0.73-0.94)** | 0.84 (0.67; 1.06) | 0.78 (0.60; 1.01) | p>0.05, RR n. r. |
| Suadicani 2012 | CMS | Denmark | 53-74 | 16 | 3,333 | 1429 | Low vs. high | 1.11 (0.97-1.27) | - | - | - |
| Goyal 2013 | NHANES III  (1988-1994) | USA | ≥ 20 | 14.2 | 16,008 | 4,225 | Low vs. high | **1.27 (1.09-1.47)** | 1.20 (0.97-1.50) | 1.16 (0.84-1.62) | - |
| Alehagen 2016 | - | Sweden | 77.7 ± 3.7 | 6.9 | 668 | 122 | Low vs. high | **1.43 (1.02-2.00)** | **1.56 (1.03-2.36)** | p>0.05, RR n. r. | - |
| Giovannini 2018 | IlSIRENTE | Italy | 85.8 ± 4.8 | 10.0 | 347 | 248 | Low vs. high | **1.41 (1.07-1.86)** | - | - | - |
| Shi 2021 | DF-TJ | China | 64.9 ± 7.5 | 9.8 | 6,155 | 876 | Low vs. high | **1.47 (1.20-1.79)** | **1.49 (1.12-2.00)** | - | - |
| Al-Mubarak 2022 | PREVEND | Netherlands | 53.6 ± 12.1 | 8.4 | 5,973 | 381 | Low vs. high | 1.21 (0.78-1.90) | - | - | - |
| Xing 2023 | NHANES  1999-2018 | USA | 51.2 ± 17.4 | 6.2 | 15,654 | 1,697 | Low vs. high | **1.32 (1.13-1.53)** | **1.82 (1.37-2.41)** | - | - |

Bold print: Statistically significant (p<0.05); Abbreviations: incr., increase; FUP, follow-up; n. r., not reported; RR, Risk ratio, SD, standard deviation.

^a^ N_deaths_ is the number of all-cause mortality cases, unless all-cause mortality was not assessed and either only CVD or cancer mortality was assessed.

^b^ Age of the cases.

^c^ Pooled result (fixed effects meta-analysis) of distinctly reported RR for men and women.
